# Characterization of the patterns of care, access, and direct cost of systemic lupus erythematosus in Brazil: findings from the Macunaíma study

**DOI:** 10.1186/s42358-024-00369-9

**Published:** 2024-04-19

**Authors:** Mirhelen Mendes de Abreu, Odirlei Andre Monticielo, Vander Fernandes, Dalianna Luise Andrade Souto Rodrigues, Cristhiane Almeida Leite da Silva, Alexandre Cristovão Maiorano, Fernando dos Santos Beserra, Flavia Rachel Moreira Lamarão, Bruna Medeiros Gonçalves de Veras, Nathalie David, Magda Araújo, Marcelly Cristinny Ribeiro Alves, Matheus Amaral Stocco, Fernando Mello Lima, Emilly Borret, Andrese Aline Gasparin, Gustavo Flores Chapacais, Guilherme Andrade Bulbol, Diogo da Silva Lima, Natália Jardim Martins da Silva, Marta Maria Costa Freitas, Blanca Elena Rios Gomes Bica, Domingos Sávio Nunes de Lima, Marta Maria das Chagas Medeiros

**Affiliations:** 1 Faculdade de Medicina, Hospital Universitário Clementino Fraga Filho, Universidade Federal do Rio de Janeiro, R. Prof. Rodolpho Paulo Rocco, 255– Cidade Universitária–, RJ 21941-617 Rio de Janeiro, Brazil; 2 MAPEAR Laboratory, Universidade Federal do Rio de Janeiro, Rio de Janeiro, Brazil; 3 Serviço de Reumatologia do Hospital de Clínicas de Porto Alegre, Universidade Federal do Rio Grande do Sul, Porto Alegre, Brazil; 4 Universidade de Cuiabá, Cuiabá, Brazil; 5 GSK, Rio de Janeiro, Brazil; 6 Faculdade de Medicina, Universidade Federal do Amazonas, Manaus, Brazil; 7 Faculdade de Medicina, Universidade Federal do Ceará, Fortaleza, Brazil

**Keywords:** Cost of illness, Disparities, Access to care, Systemic lupus erythematosus, Health inequalities, Socioeconomic factors

## Abstract

**Background:**

A cost of illness (COI) study aims to evaluate the socioeconomic burden that an illness imposes on society as a whole. This study aimed to describe the resources used, patterns of care, direct cost, and loss of productivity due to systemic lupus erythematosus (SLE) in Brazil.

**Methods:**

This 12-month, cross-sectional, COI study of patients with SLE (ACR 1997 Classification Criteria) collected data using patient interviews (questionnaires) and medical records, covering: SLE profile, resources used, morbidities, quality of life (12-Item Short Form Survey, SF-12), and loss of productivity. Patients were excluded if they were retired or on sick leave for another illness. Direct resources included health-related (consultations, tests, medications, hospitalization) or non-health-related (transportation, home adaptation, expenditure on caregivers) hospital resources.Costs were calculated using the unit value of each resource and the quantity consumed. A gamma regression model explored cost predictors for patients with SLE.

**Results:**

Overall, 300 patients with SLE were included (92.3% female,mean [standard deviation (SD)] disease duration 11.8 [7.9] years), of which 100 patients (33.3%) were on SLE-related sick leave and 46 patients (15.3%) had stopped schooling. Mean (SD) travel time from home to a care facility was 4.4 (12.6) hours. Antimalarials were the most commonly used drugs (222 [74.0%]). A negative correlation was observed between SF-12 physical component and SLE Disease Activity Index (− 0.117, *p* = 0.042), Systemic Lupus International CollaboratingClinics/AmericanCollegeofRheumatology Damage Index (− 0.115, *p* = 0.046), medications/day for multiple co-morbidities (− 0.272, *p* < 0.001), SLE-specific drugs/day (− 0.113, *p* = 0.051), and lost productivity (− 0.570, *p* < 0.001). For the mental component, a negative correlation was observed with medications/day for multiple co-morbidities (− 0.272, *p* < 0.001), SLE-specific medications/day (− 0.113, *p* = 0.051), and missed appointments (− 0.232, *p* < 0.001). Mean total SLE cost was US$3,123.53/patient/year (median [interquartile range (IQR)] US$1,618.51 [$678.66, $4,601.29]). Main expenditure was medication, with a median (IQR) cost of US$910.62 ($460, $4,033.51). Mycophenolate increased costs by 3.664 times (*p* < 0.001), and inflammatory monitoring (erythrocyte sedimentation rate or C-reactive protein) reduced expenditure by 0.381 times (*p* < 0.001).

**Conclusion:**

These results allowed access to care patterns, the median cost for patients with SLE in Brazil, and the differences across regions driven by biological, social, and behavioral factors. The cost of SLE provides an updated setting to support the decision-making process across the country.

**Supplementary Information:**

The online version contains supplementary material available at 10.1186/s42358-024-00369-9.

## Background

Systemic lupus erythematosus (SLE) has attracted attention in cost studies due to its chronic disease course, multiple co-morbidities, and long-term disability [1]. Morbidity, as assessed by measures of lupus activity, permanent organ damage, functional ability, and health status, has also been found to vary by socioeconomic status in some studies [1]. However, direct cost studies in SLE are scarce in the literature.

All resources used to treat a disease represent direct costs [2]. They usually include direct medical costs and direct non-medical costs [1]. Direct medical costs refer to the medically related inputs used to provide care, including costs associated with diagnosis, treatment, continuing care, emergency care, and rehabilitation [1, 2]. Direct non-medical costs refer to costs incurred by patients and their families that are associated with a disease but are not medical in nature, including transportation costs, household expenditures, and informal care [1, 2]. These costs are not typically included in most cost analyses but emphasize the patients’ perspective [1].

With 203 million inhabitants, Brazil is the largest country in Latin America [3]. Geographically, it is divided into 5 regions (Midwest, Northeast, North, Southeast, and South), with marked ethnic, cultural, social, economic, and healthcare standards diversity [4]. For this reason, mapping the particularities of each region’s prescriptive practice would contribute to identifying disparities in SLE management.

This study sought to assess the use of standard therapies for SLE and healthcare resource use, and to estimate the direct costs associated with patients with SLE in the 5 Brazilian regions. Additionally, the study aimed to explore factors associated with the estimated direct SLE cost.

## Methods

### Design

This cross-sectional study (Macunaíma Study or GSK Sponsor study 207353) was carried out through the application of eight questionnaires delivered during one face-to-face interview. A review of medical records for a period of 12 months (± 2 months) prior to the index date, defined by the date of the face-to-face interview, added clinical information for the analyses. No additional contact with the patients was required following the face-to-face interview [5]. The primary data collection took place between February 26, 2019 and concluded on March 6, 2020. Figure S1 presents the methodological structure of the study.

The study is inserted as a Brazilian registry of the Brazilian Society of Rheumatology. An electronic platform, called Macunaíma Software was developed for the purposes of the study and can be found through the link: www.projetomacunaima.com. Access is password hierarchical.

### Data source/data collection

The protocol included the following standard questionnaires applied during interviews with patients: American College of Rheumatology (ACR) 1997 SLE classification criteria [6]; demographic and social data assessment; clinical and laboratory data assessment; the valid Brazilian Portuguese version of quality of life 12-Item Short Form Survey (SF-12) [7]; Brazilian Economic Classification Criteria (BECC); Work Productivity Activity Index [8]; and the resources used questionnaire. The questionnaires applied in this study can be viewed in the Supplementary Material.

In this study, gender, race/color, region, and location (urban/rural and capital/other) were analyzed. The race/color variable was divided into four groups: White, Black, Asian, and multiracial. In Brazil, race/color classification is based on self-identification. Region was divided into Northeast, North, South, Southeast, and Midwest. Brazil is a country of continental dimensions and presents economic and cultural differences derived from its formation as a nation. Income and education were considered to be socioeconomic characteristics in the measurement of inequalities. Income and years of schooling were used as continuous variables in the calculation of inequality measures. Schooling was coded into five categories: 0–3, 4–7, 8–10, 11–14, and 15 or more years of education. Moreover, we considered that those who were illiterate had no schooling. For this variable, we calculated the average educational level in the range. In other words, the assumption was that within this range, people are distributed proportionally among the possible years of schooling.

Clinical and laboratory data were used to monitor disease activity by assessing predefined changes in SLE Disease Activity Index (SLEDAI) or the patient’s kidney health during the study period. Activity was assessed during the interview, 6 months before, and 12 months before the interview. Systemic Lupus International Collaborating Clinics/ACR Damage Index (SDI) score mapped damage accrual. Direct resources were categorized as (1) health-related hospital resources: consultations, tests, medications, hospitalization; (2) non–health-related hospital resources: transportation, home adaptation, expenditure on caregivers. To increase accuracy, a variety of sources were used to calculate the direct cost-of-illness (COI) [8, 9], including: the patient’s interview report, chart source, summary of hospitalization (when appropriate), prescriptions, and any other documented source of information available from the hospital. In situations where hospitalizations or complementary tests were performed in other care settings besides the study center, the resources used were captured through review of discharge summaries and accounting of reports.

For inclusion in the study, the patient needed the following: (1) a definitive classification of SLE according to the 1997 ACR criteria [6]; (2) to be ≥ 18 years of age; and (3) at least 12 months of follow-up in the same service. Those with a history of leave or retirement due to a disease other than SLE, as well as patients who refused to sign the informed consent form, were excluded. The ACR 1997 SLE classification criterion was selected because it has greater sensitivity compared to the current classification [10]. After exhaustive discussion with the study team, the decision was based on the following assumptions: (a) SLE is a syndrome and not a single disease; (b) the objective of the classification criteria is to collect a well-defined population of patients, suitable for the research, thus accepting that atypical patients are excluded; (c) specificity generally outweighs sensitivity in determining classification performance, but as a real-world study, which included a retrospective review of medical records, sensitivity would more accurately reflect the resources used in the clinical setting.

For patients’ convenience, they were recruited and selected on the day of their medical consultation. Centers were selected according to the following criteria: (1) public and tertiary outpatient facilities; (2) leading in SLE care across the region that they are located; and (3) located in a teaching hospital facility. Within this criterion, the representative services for the study were: Federal University of Manaus, for the North region; Federal University of Ceará, for the Northeast region; Federal University of Rio de Janeiro, for the Southeast region; University of Cuiabá, for the Midwest region and, finally, Federal University of Rio Grande do Sul, for the South region.

The study was assessed and approved by the Research Ethics Committee of the study’s coordinating center as well as of each participating center. Written informed consent was obtained from each patient.

### Perspective

As part of a COI study methodology, a perspective should be predefined. In our study, we selected the societal perspective since this option includes various stakeholders involved in one healthcare setting, such as the user (patient), the provider (healthcare professionals), and the payer. The patient’s perspective was captured using the patient-reported outcome (PRO) SF-12 instrument. A PRO is a subjective measure of a disease’s effect on a patient’s life. The SF-12 is a generic instrument, validated and widely used in Brazil, that has the advantage of evaluating the impact that the treatment of a disease has when compared with the treatment of other diseases. The provider’s perspective was obtained by the care practice pattern, including frequency of consultations, specialists involved, hospitalizations, and tests performed. The health system perspective was captured by information regarding access to the study site, including patients’ transportation and time to access care (e.g., consultations, exams, and treatment) [9, 11].

### Source of monetary values

Data on the values assigned to drug care at Unified Healthcare System - *Sistema Único de Saúde* (SUS) - were extracted from 3 information systems that are freely accessible online:

- Siga Brazil, administered by the Federal Senate, which discloses the government budget [12].
- Healthcare Public Budget Information System (Sistema de Informação sobre Orçamentos Públicos em Saúde [SIOPS]), administered by the Ministry of Health, which shows data from the states, federal district, and municipalities regarding revenue and healthcare expenses [13].
- Integrated System for Administration of General Services (Sistema Integrado de Administração de Serviços Gerais [SIASG]) database, a publicly available general database from the Brazilian federal government [14].

Data were extracted for all medicines that were reported during the survey. Each purchase was individually described with information captured on the drug (name, dosage form, and strength), unit purchase price, and quantity purchased in number of drug units.

### Costing

Costs associated with SLE were calculated using the following steps: (1) direct resources data collection; (2) measurement of the frequency of each resource used during the study period; (3) calculation of the cost of the resource used during the study period using the standard equation: [frequency of each resource used × monetary value of it]. Costs were categorized as health-related direct costs or non-health-related direct costs, according to the resource used. Transportation costs were included based on reports provided by patients [15–17]. The unit cost of transportation was based on the official rates of the local public transport system for each of the study sites. Home adaptation was defined as reported adjustments made to meet the needs of patients with disabilities associated with SLE (e.g., enlarging doors and adapting rooms to accommodate patient’s needs). Additionally, personal adaptations, such as the use of orthesis, were calculated and provided. Caregiving costs were collected from the patients.

### Statistical analysis

Assuming an overall recruitment number of approximately 300 patients (previous feasibility), the estimation for each center was a minimum of 50 patients. For each center, a sample size was calculated assuming a significance level of 10% and a margin of error of 10%. We used a correction for finite sample considering that each center has 130 patients, which results in a sample of 45 patients. In this case, we collected information from 60 patients per center, which was larger than the suggested sample size.

As this is a descriptive study, means, medians, standard deviations (SDs), and percentages were used to present data. Differences between regions were calculated and statistically measured to assess inequalities of lupus care in Brazil. Cost was calculated using the unit value of each resource and the quantity consumed. A gamma multivariate regression model sought to explore cost predictors in this group of patients. Statistical analyses were performed with the use of R software, and Python 3.7 was used to develop the regression model. We utilized a gamma regression model due to asymmetry in the response variable (cost). For this reason, the exponential value of the parameter estimate presents a unique and characteristic interpretation of this model.

Results were obtained by multiplying the unit cost by the number of times a resource was used in the preceding year. Each resource had its cost defined in Brazilian currency (Real [R$]) and converted into 2019/2020 US dollars, considering the average dollar values ​​in the country during the study period. To present all data about the expenditure incurred over multiple years in US dollar currency, we applied the methodology suggested by Turner et al. [18] to adjust the inflation and currency changes within health economic studies. Then the values of total cost per patient per year in local currency (Brazilian Real) were exchanged to US$, considering the exchange rate to the same period (Central Bank of Brazil conversion rates from March 31, 2019 to March 31, 2020). The average value of the US dollar quoted for the conversion of values ​​was R$4.24 for each US$1.00.

To identify possible associations between the study variables and the outcomes of interest, several statistical tests were used (according to the characteristics of the variables). Specifically, Analysis of Variance (ANOVA) or Kruskal-Wallis tests were used for group comparisons in numerical variables. In addition, the Chi-square test or Fisher’s exact test was used to measure the association between two categorical variables. Finally, Spearman’s correlation was used to measure the relationship between two numeric variables. In all tests, a significance level of 5% was used, rejecting the null hypothesis when the p-value was lower than the suggested level. For ANOVA, the null hypothesis was that the groups have equal means, and for the Kruskal-Wallis, that the mean ranks of the groups are the same, while the null hypothesis of the Chi-square and Fisher’s exact test was that there is no association between the analyzed variables. Finally, the null hypothesis of the correlation test was that the correlation between the analyzed variables was equal to zero, that is, that there was no correlation. For cases of comparisons of more than two groups according to a numeric variable, multiple comparison tests were performed with Bonferroni correction.

Regarding modeling, a variable selection process was performed based on statistical criteria (such as filling frequency), low variance, and using a forward-backward elimination variable selection method.

## Results

Of the total of 301 invited patients, one patient did not consent to the study and was excluded during the recruitment phase; a total of 300 patients were therefore included in this analysis. Patients’ sociodemographic and clinical characteristics are listed in Table 1. Mean (SD) age was 41.9 (12.8) years, and most patients were female and multiracial; however, these characteristics varied by region (*p* < 0.001).

**Table 1:** Detailed comparison of the studied population according to the distribution by region (*N* = 300)

Characteristics	Overall (*N* = 300)	Midwest (*N* = 60)	Northeast (*N* = 60)	North (*N* = 60)	Southeast (*N* = 60)	South (*N* = 60)	p-value
**Age, mean (SD)**	41.9 (12.8)	41.1 (12.5)	40.5 (10.8)	37.2 (11.5)	43.5 (14.5)	47.1 (12.4)	**< 0.001**
**Female, n (%)**	277 (92.3)	54 (90.0)	58 (98.3)	56 (93.3)	54 (90.0)	54 (90.0)	
Race/color, n (%)*							**< 0.001**
White	76 (25.3)	10 (16.7)	10 (16.7)	5 (8.3)	16 (26.7)	35 (58.3)	
Black	56 (18.7)	19 (31.7)	4 (6.7)	1 (1.7)	19 (31.7)	13 (21.7)	
Others	7 (2.3)	2 (3.3)	1 (1.7)	1 (1.7)	2 (3.3)	1 (1.7)	
Multiracial	161 (53.7)	29 (48.3)	45 (75.0)	53 (88.3)	23 (38.3)	11 (18.3)	
**Employment, n (%)**							**0.020**
Active	79 (26.3)	15 (25.0)	17 (28.3)	11 (18.3)	20 (33.3)	16 (26.7)	
Retired or sick leave due to SLE	100 (33.3)	17 (28.3)	12 (20.0)	25 (41.7)	22 (36.7)	24 (40.0)	
Unemployed	65 (21.7)	17 (28.3)	10 (16.7)	16 (26.7)	12 (20.0)	10 (16.7)	
Others	56 (18.7)	11 (18.3)	21 (35.0)	8 (13.3)	6 (10.0)	10 (16.7)	
**Marital status, n (%)**							0.398
Married or in a stable union	147 (49.0)	33 (55.0)	32 (53.3)	29 (48.3)	25 (41.7)	28 (46.7)	
Single	106 (35.3)	20 (33.3)	24 (40.0)	20 (33.3)	23 (38.3)	19 (31.7)	
Others	47 (15.7)	7 (11.7)	4 (6.7)	11 (18.3)	12 (20.0)	13 (21.7)	
**Access to care and patient journey**							
Time between the first symptoms and confirmation of the disease, years, mean (SD)	2.02 (3.70)	1.58 (2.75)	2.63 (3.85)	1.18 (1.87)	1.94 (2.96)	2.83 (5.91)	0.091
Time between onset of symptoms and start of treatment, months, mean (SD)	21.6 (39.6)	16.4 (28.2)	26.0 (35.2)	16.9 (30.2)	17.6 (24.7)	31.4 (65.6)	0.158
Travel time from home to facility, hours, mean (SD)	4.4 (12.6)	3.52 (4.3)	1.7 (1.3)	11.5 (25.4)	3.7 (8.3)	1.80 (1.7)	**< 0.001**
Missing medical appointments due to any reason, study period, mean (SD)	0.72 (1.3)	0.46 (10.9)	0.35 (0.9)	0.7 (1.1)	1.7 (1.8)	0.3 (0.8)	**< 0.001**
Number of medications per day, mean (SD)	6.59 (3.9)	3.92 (2.5)	6.0 (2.7)	6.2 (2.4)	8.5 (3.7)	8.3 (5.3)	**< 0.001**
Time between confirmation of the disease and the first visit to the rheumatologist, mean (SD)	7.00 (17.10)	8.52 (20.4)	7.92 (18.0)	4.39 (14.2)	10.12 (20.0)	3.97 (10.4)	0.213
Hospitalization, n (%)	43 (14.3)	4 (6.7)	12 (20.0)	5 (8.3)	8 (13.3)	14 (23.3)	**0.037**
Emergency unit visit, n (%)	84 (28.0)	15 (25.0)	14 (23.3)	18 (30.0)	21 (35.0)	16 (26.7)	0.636
***Schooling***							
Stopped school education due to SLE, n (%)	46 (15.3)	9 (15.0)	6 (10.0)	15 (25.0)	7 (11.7)	9 (15.0)	**0.047**
Years of schooling, years, mean (SD)	11.37 (4.65)	10.55 (3.42)	11.17 (4.74)	14.20 (3.64)	11.87 (5.59)	9.05 (4.06)	**< 0.001**
**Income status related to disease**							
Total household income, mean (SD), Brazilian currency	2656.03 (2121.94)	2463.60 (1608.02)	2220.78 (1626.55)	2697.15 (2083.41)	3160.07 (3068.51)	2717.63 (1793.36)	0.173
Loss of working days in the last 12 months, n (%)	67 (22.3)	15 (25.0)	16 (26.7)	7 (11.7)	13 (21.7)	16 (26.7)	0.24
Salary discount in the last 12 months due to SLE, n (%)	24 (8.0)	3 (5.0)	4 (6.7)	3 (5.0)	5 (8.3)	9 (15.0)	0.23
Performs paid work, n (%)	83 (27.7)	15 (25.0)	18 (30.0)	12 (20.0)	21 (35.0)	17 (28.3)	0.439
Loss of productivity related to activities of daily living, mean (SD)	5.17 (3.61)	6.22 (3.55)	5.40 (3.57)	4.87 (3.44)	4.48 (3.76)	4.90 (3.59)	0.083
Contemplated by government program due to SLE, n (%)	52 (17.3)	14 (23.3)	13 (21.7)	12 (20.0)	7 (11.7)	6 (10.0)	0.186
**Clinical and treatment variables**							
**SLE Classification Criteria (ACR, 1997), n (%)**							
Malar rash	166 (55.3)	21 (35.0)	36 (60.0)	49 (81.7)	25 (41.7)	35 (58.3)	**< 0.001**
Discoid rash	58 (19.3)	11 (18.3)	13 (21.7)	14 (23.3)	15 (25.0)	5 (8.3)	0.149
Photosensitivity	180 (60.0)	19 (31.7)	42 (70.0)	53 (88.3)	27 (45.0)	39 (65.0)	**< 0.001**
Oral ulcers	71 (23.7)	9 (15.0)	13 (21.7)	14 (23.3)	17 (28.3)	18 (30.0)	0.321
Nonerosive arthritis	221 (73.7)	24 (40.0)	52 (86.7)	53 (88.3)	48 (80.0)	44 (73.3)	**< 0.001**
Pleuritis or Pericarditis	65 (21.7)	3 (5.0)	14 (23.3)	17 (28.3)	16 (26.7)	15 (25.0)	**0.012**
Renal disorder	141 (47.0)	16 (26.7)	23 (38.3)	42 (70.0)	30 (50.0)	30 (50.0)	**< 0.001**
Neurologic Disorder	33 (11.0)	3 (5.0)	6 (10.0)	8 (13.3)	10 (16.7)	6 (10.0)	0.327
Hematologic disorder	140 (46.7)	11 (18.3)	36 (60.0)	23 (38.3)	32 (53.3)	38 (63.3)	**< 0.001**
Immunologic disorder	190 (63.3)	35 (58.3)	40 (66.7)	32 (53.3)	35 (58.3)	48 (80.0)	**0.023**
Positive Antinuclear Antibody	208 (69.3)	26 (43.3)	57 (95.0)	60 (100.0)	57 (95.0)	58 (96.7)	**< 0.001**
**Multiple morbidities, n (%)**							
Hypertension	156 (52.0)	28 (46.7)	23 (38.3)	43 (71.7)	30 (50.0)	32 (53.3)	**0.006**
Obesity	56 (18.7)	6 (10.0)	12 (20.0)	7 (11.7)	16 (26.7)	15 (25.0)	**0.059**
Smoking	30 (10.0)	9 (15.0)	2 (3.3)	0 (0.0)	5 (8.3)	14 (23.3)	**< 0.001**
Alcoholism	21 (7.0)	9 (15.0)	1 (1.7)	0 (0.0)	7 (11.7)	4 (6.7)	**0.002**
History of tuberculosis	27 (9.0)	1 (1.7)	3 (5.0)	5 (8.3)	12 (20.0)	6 (10.0)	**0.007**
**Damage accrual– SDI, n (%)**	1.42 (1.7)	0.75 (1.0)	1.53 (1.7)	1.22 (1.5)	2.62 (2.2)	1.00 (1.1)	**< 0.001**
**SLEDAI score, mean (SD)**	4.33 (5.4)	4.48 (6.1)	2.57 (4.2)	5.05 (4.8)	6.43 (6.3)	3.13 (4.4)	**< 0.001**
**Health-related quality of life (SF-12), mean (SD)**							
Physical component	37.8 (10.2)	37.6 (9.8)	37.9 (10.0)	37.5 (10.5)	38.4 (9.9)	37.8 (10.9)	0.99
Mental component	40.2 (11.2)	41.3 (11.6)	41.6 (10.2)	39.8 (10.8)	38.3 (11.2)	40.1 (12.3)	0.514
**General medicines used during the study period, n (%)**							
Folic acid	58 (19.3)	11 (18.3)	22 (36.7)	10 (16.7)	9 (15.0)	6 (10.0)	**0.003**
Antiplatelet aggregant or anticoagulant	73 (24.3)	3 (5.0)	19 (31.7)	16 (26.7)	21 (35.0)	14 (23.3)	**0.001**
Drug for delipidemia	81 (27.0)	6 (10.0)	17 (28.3)	22 (36.7)	21 (35.0)	15 (25.0)	**0.005**
Diabetes medication	16 (5.3)	1 (1.7)	7 (11.7)	2 (3.3)	5 (8.3)	1 (1.7)	**0.05**
Antihypertensive	173 (57.7)	19 (31.7)	30 (50.0)	45 (75.0)	37 (61.7)	42 (70.0)	**< 0.001**
Antimalarial	202 (67.3)	24 (40.0)	35 (58.3)	38 (63.3)	52 (86.7)	53 (88.3)	**< 0.001**
Antimicrobial	41 (13.7)	1 (1.7)	16 (26.7)	15 (25.0)	4 (6.7)	5 (8.3)	**< 0.001**
Calcium or vitamin D or anti-osteoporosis medication	175 (58.3)	7 (11.7)	42 (70.0)	48 (80.0)	46 (76.7)	32 (53.3)	**< 0.001**
Anti-digitalis	76 (25.3)	10 (16.7)	14 (23.3)	20 (33.3)	15 (25.0)	17 (28.3)	0.305
Corticoid	200 (66.7)	34 (56.7)	50 (83.3)	58 (96.7)	38 (63.3)	20 (33.3)	**< 0.001**
Gastrointestinal medicine	88 (29.3)	3 (5.0)	19 (31.7)	25 (41.7)	16 (26.7)	25 (41.7)	**< 0.001**
Thyroid hormone	29 (9.7)	3 (5.0)	10 (16.7)	4 (6.7)	4 (6.7)	8 (13.3)	0.159
Sunscreen	19 (6.3)	0 (0.0)	0 (0.0)	0 (0.0)	15 (25.0)	4 (6.7)	**< 0.001**
Psychotropic or antidepressant or anticonvulsant	101 (33.7)	8 (13.3)	28 (46.7)	18 (30.0)	16 (26.7)	31 (51.7)	**< 0.001**
Immunobiological	22 (7.3)	9 (15.0)	6 (10.0)	2 (3.3)	3 (5.0)	2 (3.3)	**0.058**
Immunosuppressant	213 (71.0)	28 (46.7)	44 (73.3)	47 (78.3)	50 (83.3)	44 (73.3)	**< 0.001**
**Immunosuppressors or biological medicines most used, n (%)**							
Hydroxychloroquine or chloroquine	222 (74.0)	33 (55.0)	42 (70.0)	39 (65.0)	53 (88.3)	55 (91.7)	**< 0.001**
Mycophenolate mofetil	90 (30.0)	2 (3.3)	15 (25.0)	27 (45.0)	29 (48.3)	17 (28.3)	**< 0.001**
Azathioprine	77 (25.7)	12 (20.0)	12 (20.0)	15 (25.0)	15 (25.0)	23 (38.3)	0.131
Methotrexate	48 (16.0)	12 (20.0)	17 (28.3)	5 (8.3)	7 (11.7)	7 (11.7)	**0.019**
Cyclophosphamide	27 (9.0)	7 (11.7)	2 (3.3)	11 (18.3)	4 (6.7)	3 (5.0)	**0.029**
Cyclosporine	6 (2.0)	0 (0.0)	0 (0.0)	2 (3.3)	4 (6.7)	0 (0.0)	**0.025**
Rituximab	11 (3.7)	1 (1.7)	4 (6.7)	3 (5.0)	2 (3.3)	1 (1.7)	0.648
Belimumab	13 (4.3)	11 (18.3)	2 (3.3)	0 (0.0)	0 (0.0)	0 (0.0)	**< 0.001**
Tacrolimus	1 (0.3)	0 (0.0)	0 (0.0)	0 (0.0)	1 (1.7)	0 (0.0)	1.000

ACR, American College of Rheumatology; SD, standard deviation; SDI, Systemic Lupus International Collaborating Clinics/American College of Rheumatology Damage Index; SFI-12, 12-Item Short Form Survey; SLE, systemic lupus erythematosus; SLEDAI, Systemic Lupus Erythematosus Disease Activity Index

Looking at associated co-morbidities, hypertension was the most prevalent comorbidity, and the highest proportion of patients with hypertension was in the North (35%). In addition, 30% of Brazilian patients with SLE were smokers and the region with the highest percentage of smokers was the South (23%). Regarding obesity, 18.7% of the Brazilian patients with SLE analyzed were obese, with the highest proportion in the Southeast (27%). In our study, the prevalence of arthritis was 73.7%, serositis was 21.7%, and cutaneous manifestations was found in 79% of the whole population. The Midwest had the lowest prevalence of arthritis (40.0%) and serositis (5.0%). The North and Northeast regions prevailed among those with cutaneous manifestations of the disease. High SLEDAI scores predominated in all regions, although disparities have been noted between regions (*p* = 0.007). Overall, at the time of the interview, the mean (SD) SLEDAI score was 4.5 (6.1), with 4.0 (5.5) in the Southeast and 5.0 (4.8) in the North. The 3 main contributors to disease activity according to SLEDAI were low complement (54 [18.0%]), arthritis (46 [15.3%]), and alopecia (45 [15.0%]). As for the clinical morbidity profile, accrual of organ damage, evaluated using the SDI, were scored for cataracts (45 [15.0%]), proteinuria (26 [8.7%]), and thrombosis (22 [7.3%]).

The mean (SD) time between first symptom onset and the start of treatment was 21.6 (39.6) months; the mean (SD) travel time from home to care facility was 4.4 (12.6) hours (Table 1). Antimalarials were the most commonly used treatment across all centers (74.0%), with the highest numbers in the Southeast (88.3%) and South (91.7%), *p* < 0.001 for all regions. Mycophenolate mofetil use was highest in the Southeast (48.3%) and North (45.0%), *p* < 0.001 for all regions. Azathioprine was used by 25.7% of all patients, with statistically similar use between centers, despite a slight predominance in the Southern region (38.9%, *p* = 0.131 for all regions). Methotrexate use was highest in the Northeast (28.3%, *p* = 0.019 for all regions), as well as the use of rituximab (6.7%, *p* = 0.648 for all regions) (Table 1).

The hospitalization rate was 21.3% in all regions, with no difference between centers (*p* = 0.651). The main reasons for hospitalization were disease activity (36 [12%]), infection (19 [6.3%]), surgery (10 [3.3%]), and morbidity management clinical (6 [2.0%]).

Overall, 15.3% of patients stopped schooling and 33.3% were retired or on sick leave due to SLE (Table 2).

**Table 2:** Total expenditure per direct resource according to the social profile of patients with SLE (*N* = 300)

Variable, n (%)	Medicine (median [IQR])	Consultation (median [IQR])	Exams (median [IQR])	Hospitalization (median [IQR])	Home adaptation (median [IQR])	Transportation (median [IQR])	Healthcare providers (median [IQR])	Caregiver (median [IQR])	Total cost, per patient/ per year (median [IQR])
**Did you stop schooling due to SLE?**									
No, 216 (72.0)	847.3 [389.9, 3595.7]	34.7 [23.1, 52.0]	117.8 [71.7, 189.0]	1007.7 [379.3, 2447.1]	23.6 [14.2, 66.0]	9.4 [5.4, 18.9]	1037.7 [283.0, 1952.8]	117.9 [31.2, 233.5]	1305.4 [610.4, 4115.4]
Yes, and did not return, 46 (15.3)	1194.9 [597.8, 4529.3]	45.2 [23.1, 69.3]	133.2 [58.0, 219.0]	861.4 [506.3, 2436.4]	35.4 [18.0, 76.7]	9.4 [5.7, 32.2]	283.0 [283.0, 636.8]	235.8 [153.3, 382.1]	2323.9 [863.8, 5453.2]
Yes, but returned, 38 (12.6)	1749.2 [541.7, 5305.0]	43.3 [28.9, 62.1]	154.6 [116.4, 221.5]	806.6 [615.1, 1646.3]	35.4 [28.3, 41.5]	9.4 [4.8, 17.0]	1250.0 [1049.5, 1450.5]	18.9 [16.5, 21.2]	2264.4 [1193.5, 5982.4]
p-value	0.037	0.151	0.076	0.966	0.943	0.457	0.488	0.158	0.008
**Employment**									
Retired or sick leave due to SLE, 100 (33.3)	1269.4 [683.0, 4190.0]	43.3 [28.9, 75.1]	132.1 [74.3, 216.4]	578.8 [324.2, 1885.9]	35.4 [14.2, 49.5]	9.4 [9.0, 23.3]	283.0 [226.4, 955.2]	70.8 [41.3, 202.8]	2148.3 [1015.8, 5356.3]
Active, 79 (26.3)	907.9 [344.9, 4312.7]	28.9 [23.1, 46.2]	119.5 [67.9, 163.8]	377.4 [342.8, 1106.7]	18.9 [14.2, 29.5]	9.4 [4.8, 17.6]	849.1 [566.0, 1132.1]	20.0 [15.3, 88.4]	1289.1 [558.0, 4545.1]
Unemployed, 65 (21.6)	590.7 [328.0, 1902.9]	37.7 [25.9, 52.0]	150.1 [79.5, 207.7]	1446.5 [739.6, 3329.9]	37.7 [21.2, 110.8]	9.0 [3.5, 18.4]	849.1 [176.9, 1556.6]	412.7 [180.4, 737.0]	1694.4 [628.0, 4186.0]
Others, 56 (18.6)	718.2 [467.2, 3083.2]	37.6 [23.1, 63.6]	117.5 [79.0, 189.2]	1464.0 [455.8, 1986.4]	94.3 [94.3, 94.3]	9.4 [5.2, 18.3]	3396.2 [1132.1, 3396.2]	112.0 [73.7, 150.4]	1111.0 [640.3, 4479.2]
p-value	0.005	0.006	0.251	0.155	0.423	0.068	0.08	0.374	0.032

IQR, interquartile range; SLE, systemic lupus erythematosus

We identified a negative correlation between the SF-12 physical component and the following parameters: SLEDAI (− 0.117, *p* = 0.042); SDI (− 0.115, *p* = 0.046); number of medications per day for multiple co-morbidities (− 0.272, *p* < 0.001); number of drugs per day specific for SLE (− 0.113, *p* = 0.051); and lost productivity (− 0.570, *p* < 0.001). For the mental component, we identified a negative correlation between the number of medications per day for multiple co-morbidities (− 0.272, *p* < 0.001); number of medications per day specific for SLE (− 0.113, *p* = 0.051); and nonattendance at appointments (− 0.232, *p* < 0.001).

Regarding the distribution of expenditure by resource item in relation to social and demographic aspects, disparities were observed according to race/color, history of school dropout due to the disease, occupational status, and social status (Tables 2 and 3). The highest total expenditure was observed among White patients (76 [25%]), and the highest expenditure on medication was among patients of Black race (56 [18.6%]). Expenditure on medication was highest among those who dropped out of school due to SLE (but returned; 38 [12.6%]) and among those on leave or retired due to SLE (100 [33.3%]). Median (interquartile range [IQR]) use of complementary exams among single patients (163.4 [83.6, 213.1]) was significantly higher versus married (or in a stable union) patients (117.8 [70.3, 183.5]) and others (105.0 [60.8, 165.6], *p* = 0.014; Table 3).

**Table 3:** Expenditure per direct resource according to demographic and economic profile of patients with SLE (*N* = 300)

Variable, n (%)	Drugs (median [IQR])	Consultation (median [IQR])	Exams (median [IQR])	Hospitalization (median [IQR])	Home adaptation (median [IQR])	Transportation (median [IQR])	Services (median [IQR])	Caregiver (median [IQR])	Total cost, per patient/ per year (median [IQR])
Race/color, n (%)*									
White, 76 (25.0)	997.6 [498.4, 766.3]	30.1 [23.1, 52.0]	132.8 [92.5, 191.9]	853.2 [405.7, 1858.0]	14.2 [14.2, 35.4]	9.4 [9.0, 18.9]	1202.8 [1061.3, 1662.7]	35.4 [21.2, 126.2]	1916.2 [702.8, 4175.2]
Black, 56 (18.6)	1086.2 [309.4, 496.5]	40.4 [28.9, 76.6]	123.6 [48.1, 240.2]	1231.9 [520.3, 2220.3]	35.4 [14.2, 96.7]	9.4 [8.3, 17.9]	2264.2 [1285.4, 2971.7]	47.2 [31.8, 613.2]	1803.9 [577.0, 5505.4]
Multiracial, 161 (56.6)	902.4 [451.5, 4015.6]	34.7 [23.1, 57.8]	123.8 [72.8, 189.6]	1007.7 [381.2, 2872.2]	38.4 [15.9, 66.0]	9.0 [3.9, 18.9]	424.5 [283.0, 866.7]	188.7 [129.7, 382.1]	1401.2 [679.6, 4559.2]
Others, 7 (2.3)	718.9 [295.2, 456.1]	40.4 [17.3, 84.0]	71.0 [56.1, 160.1]	118.2 [108.5, 358.1]	28.3 [21.2, 37.7]	18.9 [9.2, 38.6]	353.8 [353.8, 353.8]	NA [NA, NA]	1356.3 [647.3, 2103.2]
p-value	0.698	0.131	0.564	0.209	0.637	0.094	0.27	0.356	0.764
**Marital status**									
Others, 47 (15.6)	1142.1 [477.5, 312.7]	34.7 [23.1, 57.8]	105.0 [60.8, 165.6]	674.2 [439.7, 1286.5]	35.4 [14.2, 96.7]	9.4 [5.0, 10.1]	849.1 [566.0, 1132.1]	47.2 [29.5, 141.5]	1530.0 [686.2, 4555.4]
Single, 106 (35.3)	949.3 [449.1, 106.8]	40.4 [28.9, 63.6]	163.4 [83.6, 213.1]	1456.7 [514.0, 3221.4]	30.2 [14.2, 59.0]	9.4 [5.2, 23.5]	353.8 [283.0, 943.4]	35.4 [20.0, 129.7]	1752.4 [726.3, 5504.2]
Married^†^, 147 (49)	852.5 [432.9, 976.2]	34.7 [23.1, 57.8]	117.8 [70.3, 183.5]	632.2 [350.4, 1871.4]	31.8 [15.9, 47.2]	9.4 [5.9, 19.7]	990.6 [283.0, 1952.8]	283.0 [112.0, 559.0]	1517.3 [640.1, 4386.4]
p-value	0.796	0.266	0.014	0.106	0.911	0.518	0.644	0.147	0.455
**BECC**									
A, 3	1582.1 [869.7, 730.1]	46.2 [31.8, 72.2]	140.1 [96.6, 177.6]	NA [NA, NA]	NA [NA, NA]	9.4 [9.4, 63.7]	1650.9 [1650.9, 1650.9]	NA [NA, NA]	2164.5 [1927.7, 3142.2]
B1, 8	515.1 [406.1, 562.9]	23.1 [15.9, 35.8]	133.0 [119.8, 155.7]	2338.2 [2338.2, 2338.2]	47.2 [47.2, 47.2]	9.2 [7.7, 23.2]	934.0 [551.9, 1316.0]	528.3 [528.3, 528.3]	1020.7 [751.1, 1569.9]
B2, 49	1472.4 [483.6, 054.2]	28.9 [23.1, 40.4]	106.2 [71.3, 174.0]	1594.2 [1090.5, 1873.8]	35.4 [30.1, 94.9]	9.4 [4.5, 18.6]	1132.1 [707.5, 2547.2]	153.3 [88.4, 218.2]	1694.4 [675.9, 4570.0]
C1, 82	1033.3 [481.6, 706.5]	40.3 [23.1, 65.0]	116.2 [82.1, 183.5]	880.4 [555.9, 1822.3]	38.4 [24.8, 49.5]	9.4 [7.1, 25.9]	353.8 [283.0, 1132.1]	212.3 [188.7, 501.2]	1839.7 [646.1, 5096.4]
C2, 107	906.0 [360.5, 961.1]	37.0 [28.9, 63.6]	135.5 [70.2, 204.1]	850.1 [381.5, 2914.1]	23.6 [14.2, 96.7]	9.4 [7.3, 20.9]	955.2 [212.3, 2441.0]	18.9 [15.3, 27.1]	1900.0 [681.8, 5696.7]
D-E, 51	896.9 [418.1, 068.6]	40.4 [23.1, 63.6]	147.8 [79.5, 214.6]	626.5 [297.5, 1358.3]	14.2 [14.2, 19.2]	9.0 [3.5, 12.6]	212.3 [176.9, 247.6]	47.2 [16.5, 70.8]	1289.1 [657.6, 3070.5]
p-value	0.229	0.195	0.408	0.726	0.562	0.168	0.354	0.091	0.406

*Self-identified by patients; categorized according to Instituto Brasileiro de Geografia e Estatística guidelines; ^†^or in a stable union. BECC, Brazilian Economic Classification Criteria; IQR, interquartile range; SLE, systemic lupus erythematosus

Correlating total expenditure according to social, clinical, quality of life, and access profile, statistically significant disparities (correlation, p-value) were noted among schooling years (0.152, *p* = 0.008), income (0.143, *p* = 0.032), SLEDAI 12 months (0.152, *p* = 0.008), SDI 12 months (0.192, *p* < 0.001), SDI score (0.186, *p* < 0.001), SDI interview (0.260, *p* < 0.001), loss of productivity (0.110, *p* = 0.058), physical component of SF-12 (− 0.159, *p* = 0.006), missing appointments (0.123, *p* = 0.033), total number of medications per day (0.393, *p* < 0.001), and glucocorticoid use (0.204, *p* = 0.004) (Table 4).

**Table 4:** Correlation between the social, clinical, quality of life, and access profile and expenditure (*N* = 300)

Variable	Drugs (correlation [p-value])	Consultation (correlation [p-value])	Exams (correlation [p-value])	Hospitalization (correlation [p-value])	Total cost, per patient/ per year (correlation [p-value])
Schooling, years	0.110 (0.06)	-0.098 (0.114)	-0.026 (0.655)	0.091 (0.477)	0.152 **(0.008)**
Income	0.155 (0.023*)	-0.046 (0.525)	0.105 (0.122)	0.018 (0.904)	0.143 **(0.032)**
SLEDAI 12 months	0.173 (0.003*)	0.202 (< 0.001*)	0.145 (0.013*)	-0.082 (0.52)	0.152 **(0.008)**
SLEDAI interview	0.069 (0.241)	0.188 (0.002*)	0.059 (0.313)	0.162 (0.2)	0.109 (0.061)
SDI 12 months	0.202 (< 0.001*)	0.344 (< 0.001*)	0.180 (0.002*)	-0.181 (0.152)	0.192 **(< 0.001)**
SDI score	0.200 (< 0.001*)	0.339 (< 0.001*)	0.168 (0.004*)	-0.204 (0.106)	0.186** (< 0.001)**
SDI interview	0.204 (< 0.001*)	0.405 (< 0.001*)	0.177 (0.002*)	0.002 (0.985)	0.260** (< 0.001)**
Loss of productivity*	0.005 (0.937)	0.094 (0.127)	0.056 (0.345)	0.211 (0.094)	0.110 (0.058)
12-Item Short Form Survey (Physical component)	-0.087 (0.137)	-0.198 (0.001*)	-0.115 (0.05)	-0.019 (0.88)	-0.159 **(0.006)**
12-Item Short Form Survey (Mental component)	-0.016 (0.783)	-0.116 (0.059)	0.036 (0.542)	-0.118 (0.354)	-0.079 (0.174)
Missing scheduled medical appointments	0.039 (0.512)	0.248 (< 0.001*)	0.049 (0.402)	-0.028 (0.83)	0.123 **(0.033)**
Number of total medicines administered daily	0.411 (< 0.001*)	0.376 (< 0.001*)	0.338 (< 0.001*)	-0.027 (0.835)	0.393 **(< 0.001)**
Glucocorticosteroids user (n)	0.257 (< 0.001*)	0.075 (0.322)	0.009 (0.905)	-0.038 (0.79)	0.204 **(0.004)**

*Evaluated by WPAI. SDI, Systemic Lupus International Collaborating Clinics/American College of Rheumatology Damage Index; SLE, systemic lupus erythematosus; SLEDAI, SLE Disease Activity Index; WPAI, Work Productivity Activity Index. *p*-values in bold text denote statistically significant differences (*p* < 0.05)

The mean (IQR) total cost for SLE in Brazil was US$3,123.53 ($1,628.81, $4,625.81) per patient per year, with a median (IQR) cost of US$1,618.51 ($678.66, $4,601.29) per patient per year (Table 5). We have further analyzed cost and healthcare resource data according to region, with results displayed in Table S1. According to the regression model (Table 6), mycophenolate usage was the greatest contributor to SLE costs, significantly increasing costs by 3.664 times (*p* < 0.001). As for disease activity, for those with a permanent SLEDAI score between 2 and 6, when compared with the other score categories of this indicator, there is a decrease in cost of 0.756 times (*p* = 0.012). Regarding the SF-12 physical component index, it was found that an increase of 1 unit on the SF-12 score reduces the average total cost by 0.793 times. Examinations for urea or creatinine, liver or heart function also significantly contributed to increased SLE costs (1.317 times, *p* = 0.013). In addition, inflammatory monitoring (erythrocyte sedimentation rate or C-reactive protein) reduced expenditure by 0.381 times (*p* < 0.001). For details, see Table 6.

**Table 5:** Cost of SLE (US$), per patient per year, according to geographic region and resource consumed

Resources used	Overall	Midwest (60)	Northeast (60)	North (60)	Southeast (60)	South (60)	p-value
Medicine (median [IQR])	910.62 [460; 4033.51]	312.8 [119.4; 937.6]	694.6 [462.4; 3712.8]	1334.5 [615.5; 4391.8]	1751.2 [654.2; 5141.5]	950.7 [489.0; 3336.3]	< 0.001
Hospitalization (median [IQR])	900.60 [382.10; 2106.41]	1891.1 [382.4; 4194.9]	430.9 [377.4; 1858.0]	1007.7 [673.0; 1182.9]	1836.0 [598.0; 3145.1]	711.2 [475.5; 1395.1]	0.29
Caregiver (median [IQR])	849.06 [283.02; 235.85]	NA [NA; NA]	283.0 [212.3; 1981.1]	424.5 [254.7; 583.7]	1202.8 [336.1; 1981.1]	1132.1 [1061.3; 1273.6]	0.336
Exams (median [IQR])	130.85 [73.70; 191.58]	38.3 [18.5; 74.8]	153.7 [117.2; 207.2]	102.6 [59.9; 174.5]	153.1 [97.6; 189.9]	187.4 [123.4; 229.4]	< 0.001
Home adaptation due to SLE (median [IQR])	70.75 [23.58; 235.85]	1179.2 [1179.2; 1179.2]	NA [NA; NA]	235.8 [188.7; 528.3]	188.7 [117.9; 202.8]	21.2 [15.9; 35.4]	0.02
Aids and support for disability (median [IQR])	35.38 [14.15; 51.89]	82.5 [44.2; 121.5]	28.7 [16.1; 40.0]	18.9 [15.3; 40.1]	47.2 [14.2; 94.3]	14.2 [14.2; 14.2]	0.086
Consultation (median [IQR])	34.67 [23.11; 63.56]	23.1 [17.3; 34.3]	40.4 [28.9; 59.2]	34.7 [23.1; 44.0]	63.6 [37.6; 87.3]	28.9 [17.3; 52.0]	< 0.001
Transportation (median [IQR])	9.43 [5.42; 18.87]	9.4 [7.9; 18.3]	7.9 [3.3; 10.1]	6.8 [3.5; 19.1]	9.4 [9.4; 42.5]	9.4 [9.0; 18.9]	< 0.001
**Total cost, per patient per year (median [IQR])**	**1618**.**51**[**678.66**; **4601**.**29**]	**545.3** [**193.0**; **3246**.**3**]	**1334**.**1** [**645**.**1**; **4234**.**0**]	**2569.0** [**927**.**7**; **4559**.**3**]	**4036**.**6** [**1225**.**5**; **5971**.**3**]	**1556.5** [**706**.**6**; **3551**.**7**]	**< 0.001**

*Sources of values* 1. Brazilian health information system. Available from: http://www2.datasus.gov.br/datasus/index.php? area=02. Accessed February 12, 2021

2. Agência Nacional de Vigilância Sanitária (ANVISA). Available from: https://www.gov.br/anvisa/pt-br/assuntos/medicamentos/cmed/compras-publicas/lista-de-precos-maximos-para-compras-publicas. Accessed February 12, 2021

IQR, interquartile range; NA, not applicable; SLE, systemic lupus erythematosus

**Table 6:** Regression model to assess the factors that influence the total cost per patient per year

	Coefficient	SE	z	P>|z|	[0.025	0.975]	Exponential of the coefficient
Medications: Methotrexate	-0.312	0.138	-2.261	0.024	-0.582	-0.041	0.732
Medications: Mychophenolate	1.299	0.112	11.579	0.000	1.079	1.518	3.664
Exams: Liver function tests	0.275	0.110	2.491	0.013	0.059	0.491	1.317
Lupus classification: Arthritis	-0.524	0.111	-4.733	0.000	-0.741	-0.307	0.592
Accrual damage: SDI	0.163	0.052	3.119	0.002	0.061	0.266	1.177
Exams: Erythrocyte sedimentation rate (ESR) or C-reactive protein	-0.964	0.219	-4.400	0.000	-1.394	-0.535	0.381
Medications: Antihypertensive or antidigitalis or antianginal medicines	0.623	0.131	4.769	0.000	0.367	0.879	1.864
HRQoL: SF-12 physical component	-0.232	0.048	-4.884	0.000	-0.326	-0.139	0.793
Medical history: Hypertension	-0.246	0.125	-1.977	0.048	-0.491	-0.002	0.782
Schooling (years)	0.154	0.048	3.189	0.001	0.059	0.248	1.166
BECC (Criterion 2)	0.350	0.096	3.628	0.000	0.161	0.538	1.418
Medical appointment: Nursing	0.381	0.172	2.207	0.027	0.043	0.719	1.463
Exams: Transthoracic echocardiogram or transesophageal echocardiogram	0.286	0.124	2.304	0.021	0.043	0.529	1.331
Exams: Urea or creatinine test	0.849	0.260	3.270	0.001	0.340	1.358	2.337
Exams: Echo Doppler carotid arteries or subclavian or lower members	0.69	0.195	3.425	0.001	0.286	1.052	1.952
Lupus classification: Hematological manifestations	0.264	0.096	2.753	0.006	0.076	0.451	1.302
Time between initial symptoms and initial treatment (months)	0.181	0.049	3.728	0.000	0.086	0.277	1.199
Time from symptom onset to first consultation with rheumatologist (years)	-0.140	0.047	-2.986	0.003	-0.232	-0.048	0.869
SDI: Cataract	-0.290	0.137	-2.115	0.034	-0.558	-0.021	0.748
Caregiver cost	0.530	0.174	3.042	0.002	0.188	0.871	1.699
SLEDAI score: 2–6	-0.280	0.111	-2.518	0.012	-0.497	-0.062	0.756
Exams: Myocardial injury biomarker	0.350	0.152	2.307	0.021	0.053	0.646	1.418
Intercept*	7.032	0.220	32.034	0.000	6.602	7.462	1132.519

*The constant term in regression analysis and part of model building. SLEDAI 2–6: compared to the other SLEDAI categories, a score of 2–6 reduces the cost by US$1,699. Having a cataract has a lower cost compared to someone who does not have a cataract. SF-12: an increase of 1 unit in the SF-12 score reduces the average total cost by 21%. BECC, Brazilian Economic Classification Criteria; HRQoL, health-related quality of life; SDI, Systemic Lupus International Collaborating Clinics/American College of Rheumatology Damage Index; SE, standard error; SF-12, 12-Item Short Form Survey; SLEDAI, Systemic Lupus Erythematosus Disease Activity Index

## Discussion

This is the first Brazilian nationwide study that addresses patterns of care, resources used, direct cost, and loss of productivity of patients with SLE at outpatient clinics. To date, this is the most extensive assessment of COI in patients with SLE in Latin America. The relevance of the present study relies on the primary data collection, the assessment of loss of productivity (rarely found in the literature), the substantial number of patients surveyed, which is representative of the Brazilian population, and the multivariate analysis performed. The study mapped the cost of SLE in Brazil, taking into consideration Brazil’s diversity in multiple dimensions, which enhances the generalizability of our findings. We estimated more precisely the costs of SLE in Brazil and indicated that the costs tend to be the cause and consequence of clinical parameters, access to care, and behavioral factors. Characterizing the cost associated with SLE for prescriptive practices helps to elucidate the social burden of a disease that has multiple co-morbidities [19]. This study identified that the mean total cost per patient with SLE per year is US$3,123.53, with disparities both in terms of the direct cost itself and the resource items responsible for this total cost.

SLE is a chronic, relapsing-remitting, multisystemic autoimmune inflammatory disorder that predominantly affects women of childbearing age [1, 6, 20]. The clinical course and long-term damage associated with SLE, as well as the reduced life expectancy of patients with this condition, have been extensively characterized. In addition, studies have emphasized the socioeconomic and psychosocial impact of SLE [1, 20], although the monetary cost of caring for patients with the disorder has only been evaluated in a modest number of studies and a restricted number of countries [2, 15, 21–25] SLE has a negative impact on quality of life and is associated with high healthcare costs and significant productivity loss, considering that patients with SLE are generally young [2, 20]. Our study explored factors associated with increased cost of SLE, including long disease duration, high disease activity and damage, poor physical and mental health, and high education and employment levels. Several of these factors, including high disease activity and organ damage, poor physical health, and lack of family and social support, have also been shown previously to be associated with poor health-related quality of life (HRQoL) [20, 26–28]. SLE incurs a great burden on both the patient and society [20].

We identified a marked variability of health states between centers which, in turn, are also significantly correlated with the total cost of SLE. The interpretation of these findings moves us to reflect on the importance of the social aspect in determining health status [29]. This does not mean replacing a biological explanation for another purely social, but rather highlights the overlap of biological, psychological, and social phenomena as determinants of health [25, 30]. For this reason, the disparity in clinical features, patterns of care, and distribution of resource items associated with the expenditure deserve combined reflection. The impact of the historical formation of Brazil on the access of care can occur through both ethnic diversity and social inequality. These cultural and economic differences result in a lack of social cohesion that negatively affects the quality of care and access to care across the country.

It is important to emphasize that in Brazil we do not use “ethnicity” terminology, according to Brazilian Institute of Geography and Statistics (IBGE, as for its name in Portuguese). Race/color was defined by epistemological, statistical, political and Macunaíma Study methodological concerns. In Brazil, the recognition of race/color is self-perceived, being identified by self-declaration. In the Macunaíma Study, the question regarding race/color was covered in the Brazilian guidelines and referred to self-declaration. From a demographic point of view, in Brazil, there is the Afro-descendant terminology, recognized by IBGE as a grouping between multiracial groups and those individuals who identify themselves as “Black“ or of Black African ancestry, especially in the Amazon region. However, the contingent of patients who considered themselves as “Black” in this study was a self-declaration response. We had no Afro-descendant response, African Ancestry or similar. Thus, the terminology “Black” is the appropriate one for the context of Macunaíma Study as it refers to Black Brazilians, with Latin miscegenation, as the respondents declared themselves [31].

In quantitative terms, the health disparities (represented in this study by the number of consultations, exams, medications, time from home to care facility, etc.) between the different centers in this study is striking. Disparity regarding the type of resource used and socioeconomic factors was evidenced in this study. It was observed that the use of the resource “complementary exams” predominated in the South region (*p* < 0.001) and among single people (*p* = 0.014). There was also a disparity between type of drug and employment (*p* = 0.005), and type of drug and marital status (*p* = 0.014). Additionally, there was an association between the use of medication and schooling (*p* = 0.037).

While obtaining care illustrates a given individual’s behavior towards the disease, determined by sociocultural, biological, and psychological reasons, this behavior is also limited by capital and, consequently, by access [32]. In this study, we used 3 surrogates as access proxies: (1) time between onset of symptoms and start of treatment; (2) travel time from home to facility; (3) missing medical appointments due to any reason in the study period; and (4) number of medications per day. Among surrogates, we identified missing medical appointments (0.123, *p* = 0.033) and number of medicines taken per day (0.393, *p* < 0.001) as key drivers of total expenditure. Although travel time from home to facility was not significantly correlated with cost, we found that this item was strongly correlated with BECC (*p* < 0.001), which considers education, family income, and consumption power factors (Table 6). The issues of transport, access, and quality of care have been heavily studied in different scenarios in rheumatology, with significant results [15, 20, 23, 25]. From the results of this current analysis, we suggest that predictors of SLE costs are potentially amenable to psychological or social interventions and may be modified by determinants of direct costs, thereby improving patient outcome while simultaneously reducing disease costs.

From a clinical point of view, we demonstrated that disease activity, damage accrual, and glucocorticoid use were associated with an increase in the mean total cost. On the other hand, the physical and mental components of the SF-12 score were negatively correlated with the total cost (Tables 4 and 5). The relationship between use of resources, access to these resources, and consequences on clinical indicators—represented here by health status (disease activity, accrual of organ damage, and PROs)—reflects the quality of care. It remains to be seen whether the resources being allocated to care translate into better clinical outcomes.

To further our analysis, a regression model was performed to assess factors that influence the total cost per patient per year. Overall, medication, hospitalization, and caregiving were the greatest contributors to SLE cost. Additionally, disease classification parameters, activity, chronicity, multiple co-morbidities, quality of life, and access to care were also associated with the total cost per patient per year.

In addition to explaining the disparities of the disease, the set of results and explanatory models in this study lead to important questions regarding the different possible orientations in determining health policies and the inclusion of technologies in healthcare system. Our study sought to understand the cost of the disease by mapping the patient’s journey. The term “patient’s journey” refers to the evolution of the disease on the physiological plane and the complete organization of the work developed to follow this course, as well as the repercussions that this work and its organization provoke in the lives of everyone involved [33]. Several studies have attempted to elucidate SLE costs among different subgroups of patients with different organ involvement. Some studies estimated direct medical costs in patients with or without nephritis using medical claims data [34–36], one of which [34] also estimated indirect costs due to absenteeism and short-term disability; however, the number of patients with available data for this calculation was small (only 10–20 patients with nephritis).

There are limitations in this study. Patient charts, the source of complementary information in this study, were reviewed retrospectively, which can be considered a limitation since the information available from physicians’ notes in the charts may not be complete. In addition, some data obtained by interviews are susceptible to the patient’s memories and depend on the patients’ availability to answer. In this case, some data cannot be too accurate as we expected. On the other hand, such limitations were mitigated by the strategy of obtaining three sources of data collection (patient interview, medical record review and an additional one that we called “other sources”, such as prescriptions, discharge summary, test results, among others). Consistency between sources strengthen the accuracy of our data and overcomes the potential limitations highlighted. Also, this study did not assess the influence of caregivers on the cost of this illness. We realized that caregiving is a burden of SLE disease, mainly for those who care for patients with SLE with high damage index scores. However, we assumed that a protocol specifically developed to assess this issue may provide a deeper understanding of the whole picture. Herein, we included caregiver in the questionnaire, as a description of resources to be used, which may provide useful information to be explored further in a potential future study. In addition, if a home improvement took place prior to the data collection period of our study, it may not have been captured in our analysis.

Future studies should be conducted to analyze the decision-making process between therapeutic alternatives, considering the HRQoL, both in terms of micro-decision (physician–patient encounter) and macro-decision (policy choices). Such studies should also attempt to further understand the indirect costs and social consequences arising from each treatment technology [20, 32]. Long-term prospective studies should be encouraged to monitor the costs and psychosocial impact of this condition, and to better understand the factors that are associated with poor outcomes.

## Conclusion

In this COI study in SLE results suggest that average total cost per patient per year in the context of SLE in Brazil is driven by biological, social, and behavioral factors. Disparities among the five Brazilian regions may represent differences in health access or cultural behavior by the population. More studies, with longitudinal multicenter design, are necessary to address the effects of these differences in the prognosis of these patients. Our findings may have global implications as well, both for the methodological contribution and for reaching a globally representative Latin American population.

## Electronic supplementary material

Below is the link to the electronic supplementary material.

Supplementary Material 1

Supplementary Material 2

## Data Availability

The datasets used and/or analyzed during the current study are available from the corresponding author on reasonable request.

## Abbreviations

ACR: American College of Rheumatology
ANOVA: Analysis of Variance
BECC: Brazilian Economic Classification Criteria
COI: cost of illness
HRQoL: health-related quality of life
IQR: interquartile range
NA: not applicable
PRO: patient-reported outcome
R$: Brazilian Real
SD: standard deviation
SDI: Systemic Lupus International Collaborating Clinics/ACR Damage Index
SE: standard error
SF-12: 12-Item Short Form Survey
SIASG: Sistema Integrado de Administracao de Servicos Gerais
SIOPS: Sistema de Informacao sobre Orcamentos Publicos em Saude
SLE: systemic lupus erythematosus
SLEDAI: SLE Disease Activity Index
SUS: Sistema Unico de Saude
US: United States
US$: United States Dollar
Y: yes
