# Supplementary material for: Characterization of the patterns of care, access, and direct cost of systemic lupus erythematosus in Brazil: findings from the Macunaíma study
Source: Adv Rheumatol. 2024 Apr 19;64:30. doi: 10.1186/s42358-024-00369-9 (PMC13488630; doi:10.1186/s42358-024-00369-9)
Supplement: Supplementary file 1 [file 42358_2024_369_MOESM1_ESM.pdf]

Figure S1. Macunaíma project designed protocol. SLE cost of illness study in Brazil

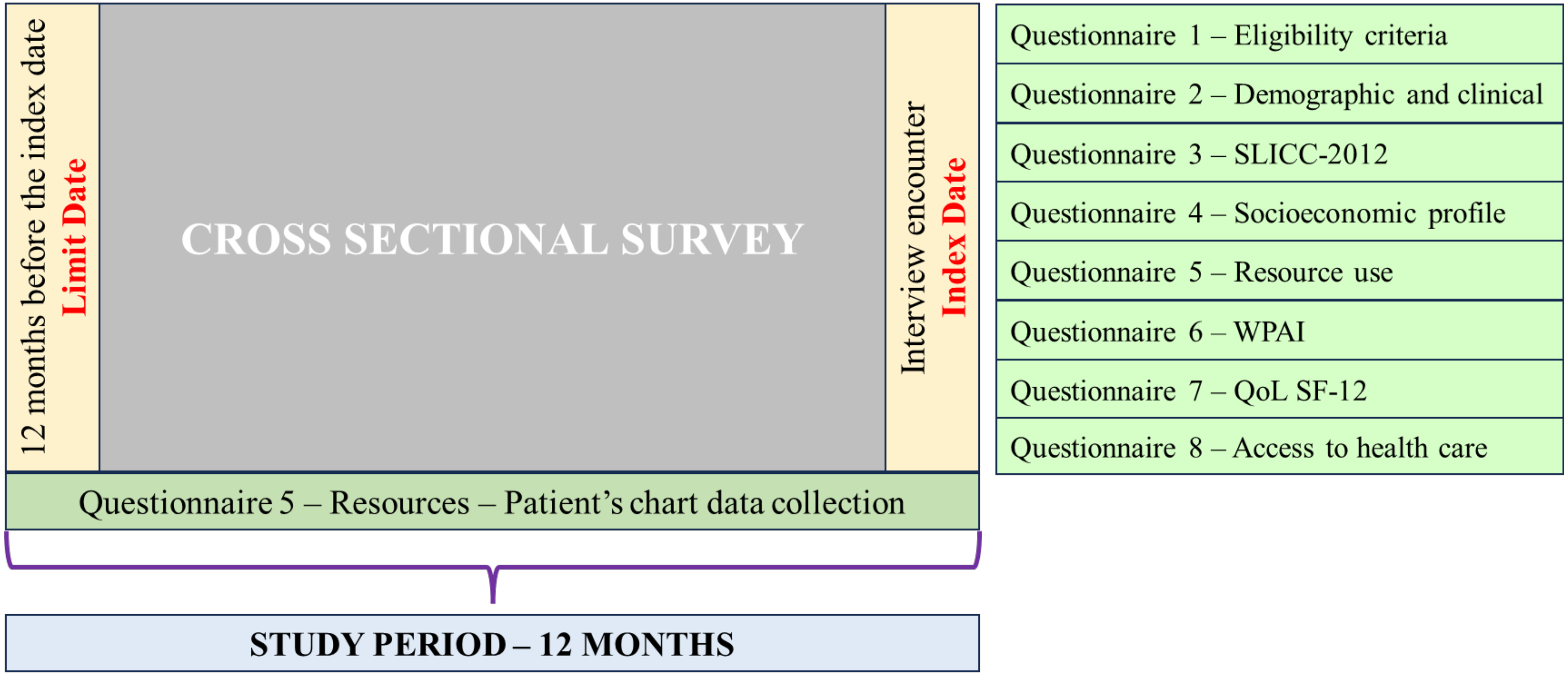

Study period: From index date to previous 12 (±2) months.

QoL, quality of life; SF-12, 12-Item Short Form Survey; SLICC, Systemic Lupus International Collaborating Clinics; WPAI, Work Productivity Activity Index.
