# Supplementary material for: Characterization of the patterns of care, access, and direct cost of systemic lupus erythematosus in Brazil: findings from the Macunaíma study
Source: Adv Rheumatol. 2024 Apr 19;64:30. doi: 10.1186/s42358-024-00369-9 (PMC13488630; doi:10.1186/s42358-024-00369-9)
Supplement: Supplementary file 2 [file 42358_2024_369_MOESM2_ESM.doc]

**Characterization of the patterns of care, access, and direct cost of systemic lupus erythematosus in Brazil: findings from the Macunaíma study**

**Running** **title:** Patterns of care and cost study of systemic lupus erythematosus in Brazil

Mirhelen Mendes de Abreu, MD, PhD^1,2^, Odirlei Andre Monticielo, MD, PhD^3^, Vander Fernandes, MD, PhD^4^, Dalianna Luise Andrade Souto Rodrigues, MD^4^, Cristhiane Almeida Leite da Silva, MD, PhD^4^, Alexandre Cristovão Maiorano, PhD^2^, Fernando dos Santos Beserra, MEng^2^, Flavia Rachel Moreira Lamarão, BSc, PhD^5^, Bruna Medeiros Gonçalves de Veras, RN, MSc^5^, Nathalie David, MD^5^, Magda Araújo, MSc candidate^5^, Marcelly Cristinny Ribeiro Alves, MD^1^, Matheus Amaral Stocco, MD^1^, Fernando Mello Lima, MD^1^, Emilly Borret, MD^1^, Andrese Aline Gasparin, MD, PhD^3^, Gustavo Flores Chapacais, BSc^3^, Guilherme Andrade Bulbol, MD^6^, Diogo da Silva Lima, MD^6^, Natália Jardim Martins da Silva, MD^7^, Marta Maria Costa Freitas, MD, MSc^7^, Blanca Elena Rios Gomes Bica, MD, PhD^1^, Domingos Sávio Nunes de Lima, MD, PhD^6^, Marta Maria das Chagas Medeiros, MD, PhD^7^

^1^Faculdade de Medicina, Universidade Federal do Rio de Janeiro, Rio de Janeiro, Brazil; ^2^MAPEAR Laboratory, Universidade Federal do Rio de Janeiro, Rio de Janeiro, Brazil; ^3^Serviço de Reumatologia do Hospital de Clínicas de Porto Alegre, Universidade Federal do Rio Grande do Sul, Porto Alegre, Brazil; ^4^Universidade de Cuiabá, Cuiabá, Brazil; ^5^GSK, Rio de Janeiro, Brazil; ^6^Faculdade de Medicina, Universidade Federal do Amazonas, Manaus, Brazil; ^7^Faculdade de Medicina, Universidade Federal do Ceará, Fortaleza, Brazil

**Corresponding author:** Mirhelen Mendes de Abreu, MD, PhD

**Email:** [mirhelen.abreu@gmail.com](mailto:mirhelen.abreu@gmail.com)

**Phone:** + 55 21 3938 2558

**Address:** Hospital Universitário Clementino Fraga Filho – Universidade Federal do Rio de Janeiro

R. Prof. Rodolpho Paulo Rocco, 255 – Cidade Universitária da Universidade Federal do Rio de Janeiro, Rio de Janeiro – RJ, 21941-617, Brazil

**Funding:** This study (GSK Study 207353) was funded by GSK.

**Supplementary Material**

1. INTRODUCTION	**4**

2. METHODS	5

2.1. Design structure	5

2.2. Standard questionnaires	5

3. RESULTS	7

3.1. Table S1. Detailed comparison of the studied population according to the distribution by region (N=300)	7

APPENDIX I – Variables and questionnaires	13

APPENDIX II – Sources of information for costs	21

# 1. INTRODUCTION

Systemic lupus erythematosus (SLE) is associated with substantial socioeconomic burden, in terms of health care resources utilization, as well as losses of productivity. The impact of SLE on the healthcare system and society has been explored by several international studies. However, cost of illness studies on SLE are limited in Brazil. To estimate the patterns of care, quality of life, resources used, and loss of productivity of patient with SLE in Brazil, this study generated valuable data.

In this Supplementary Material, we present a schematization of the methodological design, the questionnaires used for data collection, the structure of the electronic platform of the data system built for the study, the direct resources obtained, and the detailing of the study costing analysis.

# 2. METHODS

## 2.1. Design structure

This is a cross-sectional study with a retrospective assessment. The index date is defined as the date of the patient interview, and the period of the study is limited to 1 year before the index date (Figure S1).

All questionnaires were applied at the time of the interview. The review of medical records was performed to complement the information regarding the direct health resources used for patient care during the study period. In addition, the review of medical records aimed to construct the scores of disease activity at the sixth and 12th months prior to the interview, as well as the score of accumulated damage.

Figure S1. Macunaíma project designed protocol. SLE cost of illness study in Brazil

Study period: From index date to previous 12 (±2) months.

QoL, quality of life; SF-12, 12-Item Short Form Survey; SLICC, Systemic Lupus International Collaborating Clinics; WPAI, Work Productivity Activity Index.

## 2.2. Standard questionnaires

All characteristics of the questionnaires that were applied, as well as their respective objectives and more details, are described in APPENDIX 1.

**Questionnaire 1:** Eligibility criteria

**Questionnaire 2:** Demographic, clinical and laboratory for preliminary patient selection questionnaire

**Questionnaire 3:** Lupus profile questionnaire – A history of the disease

**Questionnaire 4:** Clinical status (SLE Disease Activity Index [SLEDAI], Systemic Lupus International Collaborating Clinics/American College of Rheumatology Damage Index [SDI], multiple morbidities)

**Questionnaire 5:** Resource use questionnaires (See Case Report Form [CRF] below)

a) Medicines

b) Consultations with health professionals

c) Diagnostic imaging methods

d) Diagnostic methods by clinical analysis

e) Hospital admissions

**Questionnaire 6:** Questionnaires of the socioeconomic profile

a) Brazilian economic and classification criteria

b) Social and economic questionnaire

**Questionnaire 7:** Questionnaires translated and validated for Brazilian Portuguese language and cultural

a) Health-related quality of life 12-Item Short Form Survey

b) Productivity questionnaire – Work Productivity Activity Index (WPAI)

CRF:

a) Patient’s chart source
b) Clinical data
c) Direct resources items
d) Summary of hospitalization, prescription, caregivers, and any other available documented source of information in the hospital

# 3. RESULTS

3.1. Table S1. Comparison of the direct cost and resource used according to the distribution by region (N=300)

| Region/ resource | n | **Medicine,**  median [IQR] | Consultation, median [IQR] | Exams, median [IQR] | **Hospitalization,**  median [IQR] | Home adaptation, median [IQR] | Transportation, median [IQR] | Healthcare providers, median [IQR] | **Caregiver,**  median [IQR] | Total cost, per patient per year, median [IQR] |
| --- | --- | --- | --- | --- | --- | --- | --- | --- | --- | --- |
| Midwest | 60 | 312.8  [119.4, 937.6] | 23.1  [17.3, 34.3] | 38.3  [18.5, 74.8] | 1891.1  [382.4, 4194.9] | 82.5  [44.2, 121.5] | 9.4  [7.9, 18.3] | NA  [NA, NA] | 1179.2  [1179.2, 1179.2] | 545.3  [193.0, 3246.3] |
| Northeast | 60 | 694.6  [462.4, 3712.8] | 40.4  [28.9, 59.2] | 153.7  [117.2, 207.2] | 430.9  [377.4, 1858.0] | 28.7  [16.1, 40.0] | 7.9  [3.3, 10.1] | 283.0  [212.3, 1981.1] | NA  [NA, NA] | 1334.1  [645.1, 4234.0] |
| North | 60 | 1334.5  [615.5, 4391.8] | 34.7  [23.1, 44.0] | 102.6  [59.9, 174.5] | 1007.7  [673.0, 1182.9] | 18.9  [15.3, 40.1] | 6.8  [3.5, 19.1] | 424.5  [254.7, 583.7] | 235.8  [188.7, 528.3] | 2569.0  [927.7, 4559.3] |
| Southeast | 60 | 1751.2  [654.2, 5141.5] | 63.6  [37.6, 87.3] | 153.1  [97.6, 189.9] | 1836.0  [598.0, 3145.1] | 47.2  [14.2, 94.3] | 9.4  [9.4, 42.5] | 1202.8  [336.1, 1981.1] | 188.7  [117.9, 202.8] | 4036.6  [1225.5, 5971.3] |
| South | 60 | 950.7  [489.0, 3336.3] | 28.9  [17.3, 52.0] | 187.4  [123.4, 229.4] | 711.2  [475.5, 1395.1] | 14.2  [14.2, 14.2] | 9.4  [9.0, 18.9] | 1132.1  [1061.3, 1273.6] | 21.2  [15.9, 35.4] | 1556.5  [706.6, 3551.7] |
| p-value |  | <0.001 | <0.001 | <0.001 | 0.29 | 0.086 | <0.001 | 0.336 | 0.02 | <0.001 |

# APPENDIX I – Variables and questionnaires

| **Parameters and variables** | **Questionnaires (Name)** | **Who filled the questionnaire** | **Source of information** | **Objective** |
| --- | --- | --- | --- | --- |
| **1. CLINICAL** | | | | |
| 1.1. Eligibility criteria | Questionnaire 1 – Eligibility criteria | Trained and certified researcher for the execution of the research protocol | Interview | NA |
| 1.2 Sociodemographic questionnaire | Questionnaire 2 – Questions relating to family income, personal income, sick leave due to SLE, variations in family and/or personal income due to SLE | Trained and certified researcher for the execution of the research protocol | Interview | Socioeconomic description |
| 1.3. Health status | Questionnaire 3 – Activity index, accrual damage, multiple morbidities | Trained and certified researcher for the execution of the research protocol | Interview + medical chart | SLEDAI, SDI, clinical history |
| 1.4. Brazilian socioeconomic score | Questionnaire 4 – BECC | Trained and certified researcher for the execution of the research protocol | Interview | Socioeconomic description |
| **2. DIRECT HEATH CARE RESOURCE UTILIZATION (QUALITY OF CARE CHARACTERIZATION)** | | | | |
| 2.1 Number of health professional consultations during the study period | Questionnaire 5 – Quality of care characterization | Trained and certified researcher for the execution of the research protocol | Interview + medical chart + other sources | Resource used description |
| 2.2 Health professional characterization ordered during the study period | Questionnaire 5 – Quality of care characterization | Trained and certified researcher for the execution of the research protocol | Interview + medical chart + other sources | Resource used description |
| 2.3. Number of lab exams ordered for SLE diagnosis (as required) during the study period | Questionnaire 5 – Quality of care characterization | Trained and certified researcher for the execution of the research protocol | Interview + medical chart + other sources | Resource used description |
| 2.4. Lab exams characterization ordered for SLE diagnosis (as required) during the study period | Questionnaire 5 – Quality of care characterization | Trained and certified researcher for the execution of the research protocol | Interview + medical chart + other sources | Resource used description |
| 2.5. Number of lab exams ordered for flare disease diagnosis (as required) during the study period | Questionnaire 5 – Quality of care characterization | Trained and certified researcher for the execution of the research protocol | Interview + medical chart + other sources | Resource used description |
| 2.6. Lab exams characterization ordered for flare disease diagnosis (as required) during the study period | Questionnaire 5 – Quality of care characterization | Trained and certified researcher for the execution of the research protocol | Interview + medical chart + other sources | Resource used description |
| 2.7. Number of lab exams ordered for routine clinical review during the study period | Questionnaire 5 – Quality of care characterization | Trained and certified researcher for the execution of the research protocol | Interview + medical chart + other sources | Resource used description |
| 2.8. Lab exams characterization ordered for routine clinical review during the study period | Questionnaire 5 – Quality of care characterization | Trained and certified researcher for the execution of the research protocol | Interview + medical chart + other sources | Resource used description |
| 2.9. Number of image exams ordered for SLE diagnosis (as required) during the study period. | Questionnaire 5 – Quality of care characterization | Trained and certified researcher for the execution of the research protocol | Interview + medical chart + other sources | Resource used description |
| 2.10 Image exams characterization ordered for SLE diagnosis (as required) during the study period | Questionnaire 5 – Quality of care characterization | Trained and certified researcher for the execution of the research protocol | Interview + medical chart + other sources | Resource used description |
| 2.11. Number of image exams ordered for flare disease diagnosis (as required) during the study period | Questionnaire 5 – Quality of care characterization | Trained and certified researcher for the execution of the research protocol | Interview + medical chart + other sources | Resource used description |
| 2.12. Image exams characterization ordered for flare disease diagnosis (as required) during the study period | Questionnaire 5 – Quality of care characterization | Trained and certified researcher for the execution of the research protocol | Interview + medical chart + other sources | Resource used description |
| 2.13. Number of image exams ordered for routine clinical review during the study period | Questionnaire 5 – Quality of care characterization | Trained and certified researcher for the execution of the research protocol | Interview + medical chart + other sources | Resource used description |
| 2.14. Image exams characterization ordered for routine clinical review during the study period | Questionnaire 5 – Quality of care characterization | Trained and certified researcher for the execution of the research protocol | Interview + medical chart + other sources | Resource used description |
| 2.15. Number of medicines used for comorbidity treatment (associated to SLE care, such as osteoporosis, dyslipidemia, etc.) during the study period | Questionnaire 5 – Quality of care characterization | Trained and certified researcher for the execution of the research protocol | Interview + medical chart + other sources | Resource used description |
| 2.16. Categorization (name and dosage) of medicines used for comorbidity treatment (associated to SLE care, such as osteoporosis, dyslipidemia, etc.) during the study period | Questionnaire 5 – Quality of care characterization | Trained and certified researcher for the execution of the research protocol | Interview + medical chart + other sources | Resource used description |
| 2.17. Number of specific medicines used for SLE treatment during the study period | Questionnaire 5 – Quality of care characterization | Trained and certified researcher for the execution of the research protocol | Interview + medical chart + other sources | Resource used description |
| 2.18. Categorization of specific medicines used for SLE treatment during the study period | Questionnaire 5 – Quality of care characterization | Trained and certified researcher for the execution of the research protocol | Interview + medical chart + other sources | Resource used description |
| 2.19. Number of hospitalizations during the study period | Questionnaire 5 – Quality of care characterization | Trained and certified researcher for the execution of the research protocol | Interview + medical chart + other sources | Resource used description |
| 2.20. Reasons for hospitalization during the study period | Questionnaire 5 – Quality of care characterization | Trained and certified researcher for the execution of the research protocol | Interview + medical chart + other sources | Resource used description |
| **3. DIRECT NON-HEALTH RESOURCE UTILIZATION** | | | | |
| 3.1. Types of transportation used for obtaining SLE care (health professional visits, to take exams, to take medicines, for rehabilitation, etc.) per month during the study period | Questionnaire 5 – Quality of care characterization | Trained and certified researcher for the execution of the research protocol | Interview (patient's information) | Resource used and access to care description |
| 3.2. Numbers of each type of transportation used for obtaining SLE care (health professional visits, to take exams, to take medicines, for rehabilitation, etc.) per month during the study period | Questionnaire 5 – Quality of care characterization | Trained and certified researcher for the execution of the research protocol | Interview (patient's information) | Resource used and access to care description |
| 3.3. Caregiver resource used due to SLE disease – hours required per month during the study period | Questionnaire 5 – Quality of care characterization | Trained and certified researcher for the execution of the research protocol | Interview (patient's information) | Resource used and access to care description |
| 3.4. Home adaptation due to SLE during the study period – home adaptation for home peritoneal dialysis, oxygen therapy, and so on | Questionnaire 5 – Quality of care characterization | Trained and certified researcher for the execution of the research protocol | Interview (patient's information) | Resource used and access to care description |
| **4. HEALTH-RELATED QUALITY OF LIFE** | | | | |
| 4.1. Mental health status | Questionnaire 6 – 12-Item Short Form Survey | Trained and certified researcher for the execution of the research protocol |  |  |
| 4.2. Physical health status | Questionnaire 6 – 12-Item Short Form Survey | Trained and certified researcher for the execution of the research protocol |  |  |
| **5. PRODUCTIVITY** | | | | |
| 5.1. Loss of productivity | Questionnaire 7 – WPAI score | Trained and certified researcher for the execution of the research protocol | Interview (patient's information) | Socioeconomic analysis |
| 5.2. Sick leave due to SLE during the study period | Questionnaire 7 – WPAI score | Trained and certified researcher for the execution of the research protocol | Interview (patient's information) | Socioeconomic analysis |
| 5.3. Retired due to SLE | Questionnaire 7 – WPAI score | Trained and certified researcher for the execution of the research protocol | Interview (patient's information) | Socioeconomic analysis |
| 5.4. Absenteeism due to SLE | Questionnaire 7 – WPAI score | Trained and certified researcher for the execution of the research protocol | Interview (patient's information) | Socioeconomic analysis |
| 5.5. Presenteeism due to SLE | Questionnaire 7 – WPAI score | Trained and certified researcher for the execution of the research protocol | Interview (patient’s information) | Socioeconomic analysis |
| **NOTES AND OBSERVATIONS** | | | | |
| Note 1: Other sources of information received from the patients during the interview. They were related to resources used that were not in the regular facility, such as: emergency room due to flare on another facility; hospitalization due to SLE not in the regular facility; family physician unit visit, etc. | | | | |
| Note 2: Sources of value for the resources costing: a) Siga Brazil, administered by the Senate, which discloses the government budget; b) Healthcare Public Budget Information System – SIOPS, administered by the Ministry of Health, which shows data from the States, Federal District and Municipalities regarding revenue and healthcare expenses; c) Integrated System for Administration of General Services/Sistema (Integrado de Administração de Serviços Gerais – SIASG) database. SIASG is a publicly available general database of the Brazilian federal government. | | | | |

BECC, Brazilian Economic Classification criteria; NA, not applicable; SDI, Systemic Lupus International Collaborating Clinics/American College of Rheumatology Damage Index; SLE, systemic lupus erythematosus; SLEDAI, SLE Disease Activity Index; WPAI, Work Productivity Activity Index.

# APPENDIX II – Sources of information for costs

| **Cost parameters** | **Source of information** |
| --- | --- |
| **Perspective of cost analysis: society** | |
| **1. Health direct cost** | |
| Hospitalization | SIGTAP |
| Test labs | SIGTAP |
| Image test | SIGTAP |
| Medicines for SLE | SIGTAP |
| Medicines for disabilities due to SLE | SIGA Brasil, SIOPS, SIASG |
| **2. Non-health direct cost** | |
| Cost of transportation | Patient report related to frequency of use. Values: the price of public transportation in each area during the study period |
| Cost of home adaptation | Spent on home adaptation according to patient's report during the study period |
| Cost of caregiver | Spent on caregiver according to patient's report during the study period |
| **3. Loss of productivity** | |
| Loss of productivity due to SLE | WPAI score + amount declared according to patient's report during the study period |
| **4. Predictors of direct cost of SLE** | |
| Equation performed included clinical information + SF-12 scores + resource utilization + access score. The variable of interest (outcome) was the direct cost. All variables will be initially included in the model. | According to the descriptive analysis results  Multivariate analysis. Modeling |

SF-12, 12-Item Short Form Survey; SIASG, Sistema Integrado de Administração de Serviços Gerais; SIGA, Senado Federal; SIGTAP, Sistema de Gerenciamento da Tabela de Procedimentos, Medicamentos e OPM do SUS; SIOPS, Sistema de Informação sobre Orçamentos Públicos em Saúde; SLE, systemic lupus erythematosus; WPAI, Work Productivity Activity Index.
